# Genome Analysis of *Hypomyces perniciosus*, the Causal Agent of Wet Bubble Disease of Button Mushroom (*Agaricus bisporus*)

**DOI:** 10.3390/genes10060417

**Published:** 2019-05-29

**Authors:** Dan Li, Frederick Leo Sossah, Lei Sun, Yongping Fu, Yu Li

**Affiliations:** 1 Engineering Research Center of Chinese Ministry of Education for Edible and Medicinal Fungi, Jilin Agricultural University, Changchun 130118, China; junwuzhongxin@126.com (D.L.); flsossah@gmail.com (F.L.S.); sunlei@jlau.edu.cn (L.S.); 2 International Cooperation Research Center of China for New Germplasm and Breeding of Edible Mushrooms, Jilin Agricultural University, Changchun 130118, China

**Keywords:** *Hypomyces perniciosus*, genome sequence, *Agaricus bisporus*, wet bubble disease, CAZymes, cytochrome P450 enzymes, secondary metabolites

## Abstract

The mycoparasitic fungus *Hypomyces perniciosus* causes wet bubble disease of mushrooms, particularly *Agaricus bisporus*. The genome of a highly virulent strain of *H. perniciosus* HP10 was sequenced and compared to three other fungi from the order Hypocreales that cause disease on *A. bisporus*. *H. perniciosus* genome is ~44 Mb, encodes 10,077 genes and enriched with transposable elements up to 25.3%. Phylogenetic analysis revealed that *H. perniciosus* is closely related to *Cladobotryum protrusum* and diverged from their common ancestor ~156.7 million years ago. *H. perniciosus* has few secreted proteins compared to *C. protrusum* and *Trichoderma virens*, but significantly expanded protein families of transporters, protein kinases, CAZymes (GH 18), peptidases, cytochrome P450, and SMs that are essential for mycoparasitism and adaptation to harsh environments. This study provides insights into *H. perniciosus* evolution and pathogenesis and will contribute to the development of effective disease management strategies to control wet bubble disease.

## 1. Introduction

*Hypomyces perniciosus* Magnus (syn: *Mycogone perniciosa* (Magn.) Delacr.) is an ascomycetes fungus in the family Hypocreaceae. It causes wet bubble disease (WBD) on a broad range of basidiomycetes hosts. It was first identified as a pathogen of *Agaricus bisporus* more than 100 years ago [1,2]. WBD is one of the major constraints in the commercial production of *A. bisporus*. Typical disease symptoms of WBD on *A. bisporus* include malformation of basidiome, white, fluffy mycelial growth, copious amber droplets (diseased carpophores exuding a brown malodorous liquid), and flocculent mycelia on most substrates. The infected mushrooms are unmarketable, resulting in significant yield and economic losses to the mushroom industry globally [1,3]. WBD is widespread and occurs in most countries where there is commercial production of *A. bisporus* [1,4]. The genus *Mycogone* also contains species, such as *Mycogone* sp., *M. calospora*, and *M. rosea*, which cause less severe forms of WBD compared to *H. perniciosus* [1]. Previous studies have reported variation in colony morphology and physiology [1] as well as high genetic diversity among *H. perniciosus* isolates [1,5,6].

Despite the economic importance and the considerable losses caused by the pathogen *H. perniciosus*, little is known about the genetic and pathogenic mechanisms underlying the interaction between *H. perniciosus* and the fungal host. Previous research has focused on the disease occurrence, genetic diversity, pathogenesis, pathogenicity, identification of disease resistance, and integrated control and management of the disease [3,4,7,8,9,10]. Genomic analysis of pathogens is also one of the most effective ways to obtain a full understanding of fungal pathogenesis at the molecular level [10]. Currently, many plant pathogen genomes are available, and the putative genes involved in pathogenicity have been investigated. However, the availability of mushroom pathogen genomes is still very scarce. Recently, a few genomes of mycoparasites were released, such as *Cladobotryum protrusum* [11] and *Lecanicillium fungicola* [12,13]. The genome analysis is imperative and will facilitate more rapid identification of genes associated with pathogenicity and pathogen-mushroom interactions.

In this study, we present the de novo assembled genome of *H. perniciosus*, representing the first sequenced genome in the genus *Hypomyces* using the single-molecule real-time sequencing platform (SMRT) of Pacific Biosciences (PacBio). Our specific objectives were the following: (1) to conduct high-quality genome sequencing of a highly virulent *H. perniciosus* strain HP10 and estimate its evolution time relative to species in the order Hypocreales; (2) to perform comparative analyses of genome repertoires among species in the same family. The genomic data of *H. perniciosus* will provide information regarding the genes involved in pathogenicity and can be used to assess the factors involved in host-pathogen interactions.

## 2. Materials and Methods

### 2.1. Fungal Strain

The *H. perniciosus*, strain HP10 is a brown (colony morphology), high virulent strain isolated from wet bubble-diseased *A. bisporus* at a mushroom farm in Wuhan, Hubei Province, China [3]. The identity of the pathogen was confirmed through colony morphology and microscopic analysis, Internal Transcribed Spacers (ITS) rDNA and translation elongation factor 1-alpha sequencing and by pathogenicity testing to observe characteristic symptoms of wet bubble disease on a susceptible strain of *A. bisporus* [3]. For the de novo genome sequencing of HP10, mycelial plugs of a pure culture of the strain HP10 were cultured on potato dextrose agar (PDA) plates overlaid with cellophane sheets for seven days at 25 °C under a light/dark photoperiod (12/12 h) [4]. The fungus was maintained on PDA medium at 4 °C and stored at the Engineering Research Center of Chinese Ministry of Education for Edible and Medicinal Fungi, Jilin Agricultural University (Changchun, China).

### 2.2. Genome Sequencing, Assembly, and Annotation

Genomic DNA of *H. perniciosus* HP10 was extracted from the mycelia using a CWBIOTECH Plant Extraction DNA kit following the manufacturer’s instructions (CWBiotech Corporation, Beijing, China). The genomic DNA was further assessed using agarose gel electrophoresis and was quantified using a Qubit 4.0 fluorometer (Invitrogen, Carlsbad, CA, USA). The DNA was fragmented into 20 kb using a BluePippin instrument (Sage Science, Inc., Beverly, MA, USA). The construction of a 20 kb library for HP10 was carried out according to Sossah et al. [11], followed by sequencing with a PacBio Sequel sequencer (Pacific Biosciences, Menlo Park, CA, USA) at the Engineering Research Center of the Chinese Ministry of Education for Edible and Medicinal Fungi. The *H. perniciosus* genome sequence was assembled using SMARTdenovo (https://github.com/ruanjue/smartdenovo). The assembly completeness was assessed using the Core Eukaryotic Genes Mapping Approach (CEGMA) [14] and Benchmarking Universal Single-Copy Orthologs (BUSCO) (BUSCO v1.22) [15,16]. The repeat sequences and transposable elements in *H. perniciosus* genome were identified using RepeatMasker v4.0.5 and RepeatProteinMasker [17] based on the Repbase database (http://www.girinst.org/repbase/) [18]. Tandem repeats were identified by the Tandem Repeats Finder (TRF) v4.04 (http://tandem.bu.edu/trf/trf.html) [19]. The identified repeat sequence and the transposable elements (TEs) within the genome assembly were masked with RepeatMasker [17]. Putative protein-coding genes in *H. perniciosus* genome were predicted using a combination of de novo and homology-based strategy. First, homology-based search was performed with proteomes of *Fusarium redolens*, *Fusarium oxysporum* FOX64, *Neurospora crassa*, and *Trichoderma atroviride* using Genewise [20]. Augustus v2.7 [21], GlimmerHMM v3.02 [22], Genscan v1.0 [23], and SNAP v 2006-07-28 [24] were used for the de novo prediction. GLEAN (http://sourceforge.net/projects/glean-gene) [25] was used to integrate the results from both homology-based and de novo prediction to produce a final consensus gene set. Functional annotation of *H. perniciosus* predicted genes was performed by using BLASTP (e-value of 1 × 10^−5^) to search against several protein databases such as the National Center for Biotechnology Information (NCBI) non-redundant (nr), Cluster of Orthologous Groups (COG) [26], the Gene Ontology (GO) database [27], the Kyoto Encyclopedia of Genes and Genomes (KEGG) database [28], the SwissProt database [29,30,31] the TrEMBL databases [31], and the InterPro Protein Families Database [32]. In addition, transfer RNA (tRNA), ribosomal RNA (rRNA) and non-coding RNAs (small nuclear RNA (snRNAs) and microRNAs (miRNAs) were annotated using tRNAscan-SE 1.3.1 [33], RNAmmer [34] and the Rfam database [35] respectively. The presence of mating-type idiomorph in *H. perniciosus* genome was assessed by sequence similarity searches using mating-type genes and flanking gene sequences from the order Hypocreales retrieved from NCBI database as query. The mating type sequence was searched against the NCBI’s conserved domain database [36] to find the conserved domains. The presence or absence of any gene was rechecked through mapping back the reads on these genes [37].

### 2.3. Functional Annotation of Pathogenicity-Related Genes and Secondary Metabolites

Carbohydrate-active enzymes (CAZymes), protease, transporters, and cytochrome P450s were predicted using the CAZymes database, MEROPS, Transporter Classification database, and the P450 database [38,39,40,41,42], respectively. Secretory proteins, membrane proteins, and Glycosylphosphatidylinositol (GPI)-anchored proteins were identified by SignalP 3.0 [43], TMHMM [44], and PredGPI [45], respectively. Protein localization signals were identified using TargetP [46]. The virulence-associated genes were identified using the pathogen-host interaction database (PHI) [47] and database of fungal virulence factors (DFVF) [48]. Secondary metabolites were predicted by combining antiSMASH 4.0 (https://fungismash.secondarymetabolites.org/#!/about) [49] and the NaPDoS (http://napdos.ucsd.edu) database [50].

### 2.4. Orthology and Phylogenetic Analysis

The protein sequences of *H. perniciosus* (HP) and eight other reported fungal species were first used to identify orthologous gene families using OrthoMCL (v.2.0.9) [51], including *Cladobotryum protrusum* (CP), *Clonostachys rosea* (CR), *Lecanicillium fungicola* (LF) *Metarhizium acridum* (MA), *Tolypocladium inflatum* (TI), *Trichoderma longibrachiatum* (TL), *T. reesei* (TR), and *T. virens* (TV). A total of 4043 single-copy genes were identified among the nine species. The proteins of single-copy gene families were aligned using MUSCLE [52]. The poorly aligned positions and divergent regions of each codon alignment were trimmed with Gblocks [53] and concatenated into one super gene for each species. RAxML-8.0.26 [54] was used to construct a maximum-likelihood (ML) phylogenetic tree using the LG+I+G+F amino acid substitution matrix model selected by ProtTest (v. 3.4) [55] with 1000 bootstrap replicates. The species divergence times among the species were estimated with A Markov chain Monte Carlo (MCMC)Tree module in Phylogenetic Analysis by Maximum Likelihood (PAML) v4.7a [56]. The calibration times obtained from TimeTree (http://www.timetree.org) [57]. Computational Analysis of Gene Family Evolution (CAFE) [58] was then used to identify the expansion and contraction of the orthologous gene families across these species with a stochastic birth and death model using a lambda value of 0.314, a *p*-value of 0.01, and 1000 random samples [59]. The evidence of positive selection of genes were tested using the branch-site model of CodeML in PAML [56] software by calculating the dN/dS ratio between the species in the phylogenetic tree (*p* ≤ 0.01). The False Discovery Rate (FDR) estimates were computed using the Benjamini–Hochberg procedure and FDR ≤ 0.05 were considered significant. The positively selected genes identified were annotated in PFAM, GO and KEGG databases.

## 3. Results

### 3.1. De Novo Genome Sequencing of H. perniciosus

The de novo whole-genome sequencing of *H. perniciosus* HP10 yielded 8.38 Gb of sequenced data using two SMRT cells (190 X sequence read coverage), which were assembled into 23 scaffolds (Table 1). The de novo assembled genome of HP10 was estimated to be ~44 Mb in size and contained a scaffold N50 length of 5.10 Mbp and a scaffold N90 length of 2.01 Mbp, as well as a 44.39% GC content (Table 1). Its assembly completeness was then evaluated with CEGMA and BUSCO using the fungal database. The analysis showed 98% and 99% for CEGMA and BUSCO, respectively, confirming that the HP10 genome was of high quality. This genome has been deposited into the NCBI database under accession number: SPDT00000000.

A total of 10,077 genes with an average sequence length of 1683.14 bp in *H. perniciosus* HP10 were predicted by the combination of homology-based and de novo-based gene prediction methods. Of these genes, the coding sequences with an average length of 1508.99 bp contained 2.59 exons with an average length of 583.55 bp and introns with an average length of 109.82 bp. We then functionally annotated these protein-coding genes using seven databases and found that a total of 9768 (96.93%) genes could be classified in at least one database. Among them, 10,607 (96.40%) genes were homologous in the NCBI Non-Redundant Protein Sequence Database (NR) database, followed by the TrEMBL database (9736, 96.62%), InterPro database (7112, 70.58%), Swiss-Prot database (6331, 62.83%), KEGG database (6211, 61.64%), GO database (5306, 52.65%), and Clusters of orthologous groups for eukaryotic complete genomes (KOG) database (4343, 43.10%) (Supplementary Figure S1). In addition, combining homology-based and de novo prediction of non-coding RNA (ncRNA) and repeat elements revealed HP10 contained 0.17% of ncRNA and 25.27% of repeat elements. The annotated ncRNAs included 275 transfer RNA (tRNA) genes, 223 ribosomal RNA (rRNA) genes, 31 small nuclear RNA (snRNA) genes, and 53 microRNA (miRNA) genes (Supplementary Table S1). For the annotation of the repeat elements (Supplementary Table S2), the most abundantly characterized elements were long terminal repeats (LTRs), which accounted for 14.56% of the genome, whereas DNA transposons comprised only 1.23%. The other class of retrotransposons, the non-LTR elements (short interspersed elements (SINEs) and long interspersed nuclear elements (LINEs) represented 4.06% of the assembled sequence in which nearly no SINE elements (0.005%) were detected.

### 3.2. Identification of Mating-Type Idiomorphs in H. perniciosus

We identified *MAT 1-2* locus (three copies) in *H. perniciosus* HP10 genome based on the presence of conserved domains and sequence similarities with mating-type genes of the order Hypocreales, but the MAT 1 idiomorph was not found. The three copies of *MAT1-2-1* genes in HP10 genome were located on scaffold utg16, utg81 and utg84. No flanking genes were located on scaffold utg84, however, the *MAT1-2-1* gene was flanked by *SLA* (cytoskeleton assembly control) upstream and *APN1* (DNA lyase) downstream on scaffold 81. The other flanking genes (*APN2* (DNA lyase), *APC5* (Anaphase-promoting complex subunit 5), *CIA30* (Complex I intermediate-associated protein 30), and *CoxVIa* (Cytochrome c oxidase subunit VIa)) were located upstream of the *MAT1-2-1* gene on scaffold utg16.

**Figure 1:**
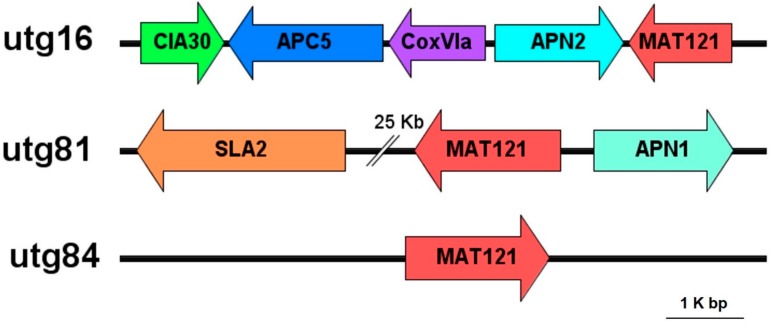
Organization of the mating-type loci (MAT) and flanking regions for in *H. perniciosus* HP10 genome. The arrows represent the orientation of the MAT1-2 genes *APC5* (Anaphase-promoting complex subunit 5), *APN1* (DNA lyase), *APN2* (DNA lyase), *CIA30* (Complex I intermediate-associated protein 30), *CoxVIa* (Cytochrome c oxidase subunit VIa) and *SLA* (cytoskeleton assembly control).

The fungus also encodes many other sex-related genes such as pheromone receptor genes, meiotically up-regulated gene, *Clr5* domain, and mitogen-activated protein kinase genes. Figure 1 shows the proposed organization of the mating-type gene structure of HP10 and *Mat1-2-1* gene structure as observed from the genome annotation.

### 3.3. Genome Evolution and Phylogenomic Analysis of H. perniciosus

**Figure 2:**
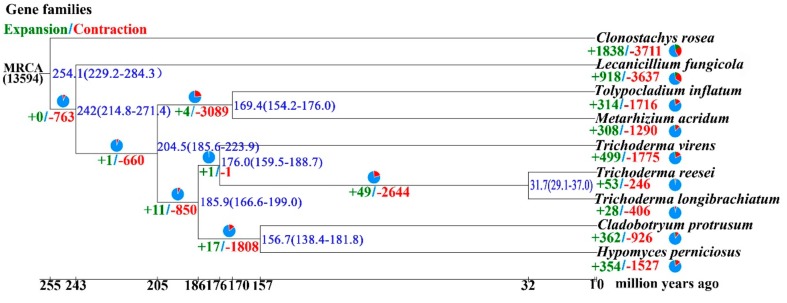
The *H. perniciosus* genome phylogeny and analysis of contraction and expansion gene families analysis among nine fungal species. The divergence time range (blue), the number of expanded (green) and contracted (red) gene families is shown at each branch. The estimated divergence time (MYA: million years ago) is shown at the bottom. MRCA: most recent common ancestor.

The proteomes of HP10 and eight other fungal species (Supplementary Table S3) in the order Hypocreales were clustered into 13,755 gene families using OrthoMCL, including entomopathogenic *Metarhizium acridum* (MA), and *Tolypocladium inflatum* (TI), mycoparasitic *C. protrusum* (CP), *Clonostachys rosea* (CR), *Lecanicillium fungicola* (LF), *T. longibrachiatum* (TL), and *T. virens* (TV), and saprotrophic, *Trichoderma reesei* (TR). A total of 4802 (34.91%) gene families were shared among all nine species. HP10 contained 963 (7.00%) unclustered genes, 63 (0.46%) unique gene families and an average of 1.1 genes per family. Overall, 4043 (38.11%) single-copy genes were identified in all nine species and were used to construct a phylogenetic tree (Figure 2). The tree placed HP and CP as sister taxa, that is closely related to *Trichoderma* spp., consistent with its classification in the family Hypocreaceae. HP is distantly related to MA, TI, LF, and CR. MA, TI, LF, and CR belong to the families Clavicipitaceae, Ophiocordycipitaceae, Cordycipitaceae and Bionectriaceae, respectively. Divergence time analysis showed that all the species diverged from a common ancestor of Hypocrealean fungi around 254.1 million years ago (MYA). HP is most closely related to CP with an estimated divergence time of ~156.7 MYA. The divergence time between *Trichoderma* species and CP and HP is estimated to be 185.9 MYA.

**Figure 3:**
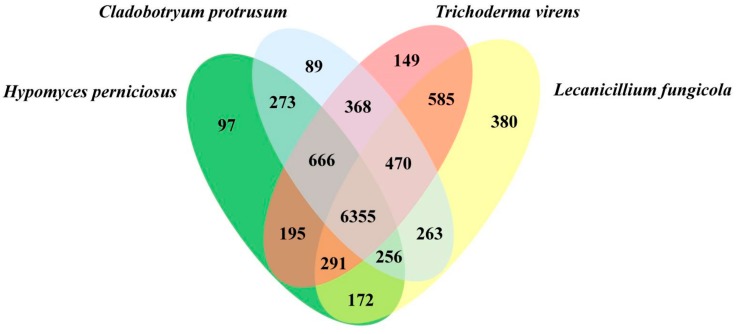
The Venn diagram shows shared and unique orthologous gene clusters among different mycoparasitic fungi (CP, HP, TV, and LF) that cause disease on *A. bisporus*.

We further identified a total of 10,609 orthologous gene clusters among the closely related Hypocreales (mycoparasites) species that cause disease on *A. bisporus*, including HP, CP, TV, and LF (Figure 3). Among them, 6355 (59.90%) gene clusters were shared among all four species, representing the ancestral gene families. In addition, 97 (0.91%), 89 (0.84%), 149 (1.41%), and 380 (3.58%) gene clusters were unique to HP, CP, TV, and LF, respectively. The numbers of orthologous gene clusters shared among HP and CP, HP and TV, HP and LF were 273 (2.57%), 195 (1.84%), and 172 (1.62%), respectively. The highest number of gene clusters 666 (6.28%) shared uniquely among three species was HP, CP, and TV. The *H. perniciosus* enriched genes were related to cellular processes, CAZymes, peptidase, and Secondary Metabolites (SMs) and showed potential for pathogenicity and adaptation to the environment.

Analysis of 5092 shared gene families based on the constructed phylogenetic tree revealed 364 (6.95%) expanded (*p* < 0.05) and 1527 (29.99%) contracted gene families in HP10 (Figure 2). Gene contraction was widespread during the diversification of Hypocreales lineages, and the largest gene family loss was observed for CR 72.88% (3711). Similarly, the Hypocreaceae lineage was characterized by the loss of gene families 16.69% (850) and the divergence of HP10 and CP 35.51% (1808). With the exception of TV 34.86% (1775) and HP 29.99% (1527), the gene family contraction was smaller in the family Hypocreaceae than in the other species (246–926). The 1527 gene family contractions calculated in HP contained 1535 (15.23%) genes, out of which only eight (8) genes were significant at *p* < 0.5. PFAM annotation revealed all the eight genes were ATP-binding cassette (ABC) transporter proteins. Similarly, the expanded gene families in HP 4.31% (354) contained 527 (5.23%) genes with 270 genes significant at (*p * < 0.5). 10.34% (28) of the significant 270 genes were genes with no known annotation in the seven different databases used for the annotation of the predicted-coding genes. PFAM annotation of the expanded genes revealed genes related to peptidases, CAZymes, SMs, and adaptation to the environment. The five most abundant expanded genes were ankyrin repeat (17), phosphorylase superfamily (14), protein kinase domain (12), BCS1 N terminal (9), and protein of unknown function (DUF3638) (9). To identify genes under positive selection, the 5092 orthologous genes from the phylogenetic analysis was used in PAML analysis [56]. The results revealed 8.3% (423 genes at *p* < 0.01) were under positive selection in *H. perniciosus* (Supplementary Table S5). Among them, 342 genes were annotated in the PFAM database. The most abundant GO terms for PSGs for biological process, cellular component and molecular function were cellular process (3 genes), membrane (12 genes) and binding (15 genes), respectively (Supplementary Figure S2). The top positively selected genes with enriched GO terms include nucleotide binding, nucleoside phosphate binding, oxidoreductase activity, oxidizing metal ions, and ferroxidase activity.

### 3.4. Carbohydrate-Active Enzyme (CAZymes) in H. perniciosus

**Figure 4:**
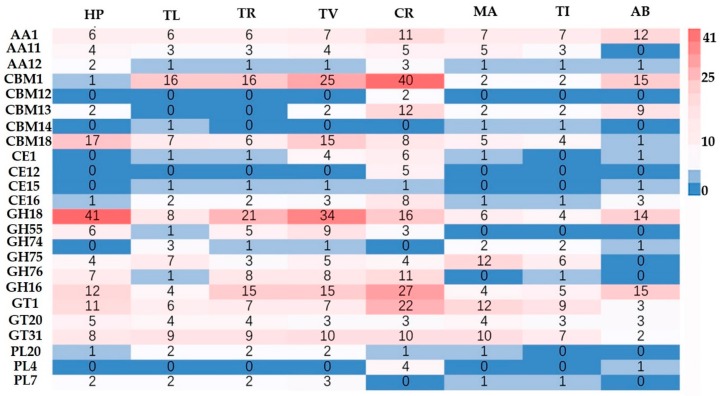
CAZyme richness of different fungi with different lifestyles (HP, TL, TV, and CR, mycoparasitic; AB (*A. bisporus*) and TR, saprotrophic; MA and TI, entomopathogenic).

The CAZymes analysis revealed the genome of HP10 contained a total of six classes: glycoside hydrolases (GH = 186), glycosyl transferases (GTs = 83), carbohydrate esterases (CEs = 6), auxiliary activities (AAs = 30), carbohydrate-binding molecule (CBMs = 28), and polysaccharide lyases (PL = 3). These diverse gene families are associated with fungal cell wall synthesis, modification, and degradation. We then used the hidden markov model (HMMs) from dbCAN2 meta server [60] to identify 119 CAZymes encoding genes in the secretome, including 12 CE, 84 GH, 3 GT, 4 PL, and 16 AA (Supplementary Table S6). Among them, the GH18, GH55, and GH75 families associated with fungal cell wall degradation are highly represented in HP10 secretome. We next compared the CAZymes number of HP with other pathogenic fungi with a different lifestyle and host mushrooms (Figure 4). The CAZymes numbers of HP10 were higher than those of the host *A. bisporus*, the entomophagous fungi (MA and TI) and saprophytic TR, but lower compared with the mycoparasitic fungi (TL, TV, and CR) (Supplementary Table S7).

Among genes encoding GH families, GH18 was the most abundant CAZymes module in HP10 compared to all of the other species analyzed. Other GH families (β-1,3-Glucanases) in HP10 involved in mycoparasitism include GH5, 16, 17, 30, 55, 64, and 81. In addition, four genes encoding GH75 (chitosanases) were found in HP10, which are known to degrade chitin [61], but were absent in the host *A. bisporus*. In contrast to the host *A. bisporus*, HP10 also lost gene-encoding cellobiohydrolases (GH6 and GH7) that are key enzymes regulating cellulose degradation and contracted nearly half the number of AA genes involved in lignin degradation. In addition, all of the fungi examined have cellulose degrading enzymes, such as GH74 with the exception of HP10 and CR. Within the family Hypocreaceae, the HP10 genome contained the highest number of CAZymes genes encoding GTs (83). The most abundant GT families in HP10 were GT1, GT31, GT90, GT32, GT8, and GT20. The number of GT2 (chitinase) was similar for all of the species analyzed. These GTs are mainly associated with chitin synthesis, cell wall biosynthesis, and glycosylation. All four Hypocreaceae fungal species possessed members of two families of putative polysaccharide lyases (PL7 and PL20) that may have activity specific to pectin [13].

### 3.5. Secondary Metabolites in H. perniciosus

The HP10 genome contained a total of 91 SMs gene clusters and 64 SM genes from the antiSMASH and NaPDoS analysis, respectively (Supplementary Tables S8 and S9). We found 14, 13, 9, and 7 clusters belonged to type 1 polyketide synthases (T1PKS), non-ribosomal peptide synthetase hybrids, terpene synthases, and non-ribosomal peptide synthetases (NRPS). The antiSMASH analysis revealed one T1PKS bikaverin biosynthetic gene cluster and four T1PKS-NRPS biosynthetic gene clusters, which are LL-Z1272beta, chaetoglobosins, betaenone (C/A) and leucinostatins, and one putative cluster, nivalenol/deoxynivalenol/3-acetyldeoxynivalenol. The NaPDoS analysis revealed 40 genes for PKS and 24 for NRPS. The top five highest pathway products for NaPDoS were: lovastatin (14), HC-toxin (10), fumonisin (9) compactin (6) and complestatin (10), and naphtopyrone (5). The majority of SMs in HP10 show genes involved in the biosynthesis of toxins, and fungus-host interaction, such as the production of antibiotics, anthelminthics, and antifungals. In addition, the high number of unknown SMs in HP10 shows the potential for the production of bioactive compounds.

### 3.6. Pathogenicity-Related Genes in H. perniciosus

We performed BLAST analysis of HP10 proteomes against the PHI gene database, Transport Classification Database (TCDB), and DFVF to find potential genes related to virulence (Supplementary Tables S10–S12). The analysis revealed that 945, 422, and 402 protein-coding genes were involved in PHI, TCDB, and DFVF, respectively, which accounted for 9.38%, 4.19%, and 3.99% of the total proteins in HP10. We also found 205 genes of DFVF in the PHI database. The genes in the PHI database were classified as a chemistry target: resistance to chemicals (3) effectors (plant avirulence determinant) (1), enhanced antagonism (2), increased virulence (hypervirulence) (26), lethal (55), loss of pathogenicity (86), reduced virulence (343), and unaffected pathogenicity (429). The HP10 genome encodes 630 secretory proteins (6.25% of the total predicted proteins) with a signal peptide. Annotation of the secretory proteins (Supplementary Table S13) showed that *H. perniciosus* secretome includes 180 PHI genes, 186 putative effector proteins (Supplementary Table S14), 119 CAZymes (Supplementary Table S6), and 191 proteases.

A number of HP10 putative PHI genes were found in the cereal pathogenic fungi *Fusarium* spp. (526 genes), *Magnaporthe oryzae* (161), *Cochliobolus* spp. (9), and *Ustilago maydis* (7). Similarly, 26 and 9 genes were similar to entomophagous fungi (*Metarhizium robertsii* (13) and *Beauveria bassiana* (13)) and necrotrophic fungi (*Botrytis cinerea* (7) and *T. virens* (2)), respectively. Interestingly, numerous HP10 genes were found in animal pathogenic fungi such as *Cryptococcus neoformans* (26) and *Candida albicans* (26). This may be due to *H. perniciosus* possessing LysM, which masks chitin on the external surface of the hyphae from being detected by the hosts. This phenomenon is present with animal fungal pathogens because they lack infection structures such as appressoria to attach to the host. In addition, we identified some genes that might be involved in breaking host physical barriers and in pathogenicity, nutrient acquisition, and adaptation to environmental stress, including proteases (399) (Supplementary Table S15), cytochrome P450 (125) (Supplementary Table S16), G protein-coupled receptors (GPCRs), hydrophobins, PacC T2 family RNases, the necrosis-inducing protein NPP1, GLEYA adhesin domain proteins, and MD-2-related lipid-recognition.

## 4. Discussion

The button mushroom (*A. bisporus*) is of socio-economic importance for human nutrition globally, despite production constraints by fungal and bacterial diseases [62,63]. *H. perniciosus* is the causal agent of the wet bubble disease that affects *A. bisporus* and other mushrooms around the world, resulting in the loss of millions of tons of mushrooms [1,3,64]. Recent advances in biotechnology have led to the availability of the whole genome sequence of edible mushrooms and pathogenic fungi [65,66,67,68]. However, the global genomics and transcriptomic studies of the interaction between *A. bisporus* and *H. perniciosus* have rarely been investigated. In this study, we used the PacBio Sequel platform to sequence the genome of *H. perniciosus*, the first reference genome for the genus *Hypomyces*. In addition, we estimated the genome evolution time, carried out a phylogenomic study on the order Hypocreales. The completeness and quality of *H. perniciosus* genome assembly are comparable to those of the *C. protrusum* genome assembly which was generated with PacBio sequel long-read sequencing technology [11]. The *H. perniciosus* genome size was within the range observed for the family Hypocreaceae (e.g., *C. protrusum* (CP), *T. virens* (TV), and *T. harzianum* (TH)) and other sequenced ascomycetes in the class Sordariomycetes [11,67]. The genome of *H. perniciosus* is composed of 25.27% repeat sequences, the largest in the family Hypocreaceae and other pathogens such as *Pyricularia oryzae* (11.75%) [69], *Valsa mali* (14.05%) [70], and *Ustilago virens* (25%) [71]. The expansion of repeat sequences is a common feature among some fungi with known large genomes, such as *Puccinia graminis* (45%) [72], *Tuber melanosporum* (58%) [73], *Blumeria graminis* (64%) [74], and *Ophiocordyceps sinensis* (74.67%) [75]. The higher number of repeat elements in *H. perniciosus* could be a result of poor or inefficient DNA removal mechanisms compared to other members in the family Hypocreaceae and may explain its relatively large genome size [75]. Previous studies have shown that the repeat sequences have an important role in driving pathogenicity variation in many pathogenic fungi and enable us to better understand the genome architecture of a pathogen and evolution [76,77].

The protein-coding genes in *H. perniciosus* (based on the genome size) were relatively low in number (10,077) compared to those in the genomes of the mycoparasite CP (11,003), TV (12,250), and TH (14,294) [11,67]. This may be due to the high number of repeat sequences in the genome. The genes are mainly located in repeat sequences poor regions, and the gene density is heterogeneous when compared with that of other ascomycetes [73]. *H. perniciosus* shares the highest number of unique orthologous gene families with *Trichoderma* (or members of the family Hypocreaceae especially the genera *Escovopsis*, *Cladobotryum*, or *Trichoderma*). Phylogenetic analysis from the total of 4043 gene families shared among all of the nine species used revealed *H. perniciosus* belonged to the family Hypocreaceae and diverged from its common ancestor ~156.7 million years ago. This divergence time is consistent with those reported by Sung et al. [78].

We identified several expanded gene families, such as different types of ankyrin repeats, glycosyl hydrolases, lipases, peptidases, transporters (ABC, MFS, sugar, ion, and phosphate), and several groups of predicted signaling genes, including kinases and transcription factors; in addition, SMs are lineage-specific genes, suggesting that important protein-coding innovation occurred in these lineages [79]. The gene family expansion in *H. perniciosus* might have contributed to its pathogenicity, host lifestyle, the wide adaptability to varied climates, and worldwide infection of *A. bisporus* and other mushrooms [80]. Moreover, gene family contraction in *H. perniciosus* was more prominent than gene family expansions. We hypothesize that these gene contractions have limited the host range of *H. perniciosus* compared to *Trichoderma* species. Previous studies also have shown the massive gene contraction in pathogens might lead to the adaption of host changes [81,82]. Fungal effectors, cell wall degrading enzymes and secondary metabolites genes are known to be under positives selection in pathogenic fungi [83]. The positively selected genes in *H. perniciosus* may have contributed to its mycoparasitic lifestyle and adaptation to a harsh environment [80,84,85]. The comparative analysis between *H. perniciosus* and the other mycoparasites that cause disease in *A. bisporus* revealed the genome structure and overlap among the orthologous clusters, which can elucidate gene function and evolution of the proteins involved in pathogenicity [80]. Identification of those genes is relevant in designing effective disease control structures that can be applied to all of the pathogens examined. Fungi are known to exhibit two different sexual lifestyles, homothallism (self-fertility) and heterothallism (self-sterility) [86]. The organization of *H. perniciosus* HP10 mating-type and flanking genes (*SLA-MAT-APN*) is consistent with other representation in Hypocreales [86]. The analysis of the mating-type structure in *H. perniciosus* HP10 genome suggests the fungus is heterothallic. *Cladobotryum protrusum* [11] and *Trichoderma reesei* [87] in the family Hypocreaceae are heterothallic and also contain the MAT 1-2 idiomorph. The three copies of *MAT1-2-1* may be due to the number of nuclei in the conidia. As of now, there is no report of mating-type idiomorph in *H. perniciosus*. The genome sequence of *H. perniciosus* has provided some insights into the mating structure of the pathogen. However, many fungi can switch between heterothallism and homothallism [85,88], therefore, additional functional characterization of the mating type genes including the identification of the flanking genes and genome resequencing of various populations of *H. perniciosus* is required to ascertain these results.

The total number of secretory proteins, CAZymes, P450, and SMs in *H. perniciosus* is smaller than that of *C. protrusum* and *Trichoderma* species. The secretory proteins and pathogenicity-related genes are crucial effectors responsible for host-pathogen interactions [89,90]. The analysis of *H. perniciosus* secretory proteins and pathogenicity-related genes shows that there is a likelihood that the fungus does not require considerably large numbers of CAZymes and SMs during host infection, but *H. perniciosus* utilizes secretory proteins, key pathogenic GHs (GH18, GH75, GH 76 GH 16), CBM50 (LysM domain), CBM18 and CE4 (chitin deacetylase), peptidases, and SMs to infect its hosts [86,91].

## 5. Conclusions

In summary, the genome analysis of *H. perniciosus* has provided insights into the evolution, pathogenicity, and genes influencing the pathogen interaction with its fungal host. The genomic resource data generated in our study can be further mined and the identified genes characterized to explain the basis of the mycoparasitic lifestyle in *H. perniciosus* and the family Hypocreaceae in general, and it can serve as the basis for developing efficient disease-management strategies to mitigate wet bubble disease.

## Figures and Tables

**Table 1:** Summary of *H. perniciosus* HP10 genome assembly and annotation statistics.

HP10	
Total scaffold number	23
Total length of sequences	44,006,492 bp
N50	5,099,276 bp
N90	2,013,553 bp
GC content	44.39%
Annotated protein-coding genes	10,077
Repeat sequences proportion	25.27%
ncRNA proportion	0.27%
CEGMA	97.98%
BUSCO	99.3%

## Supplementary Materials

Supplementary materials can be found at https://www.mdpi.com/2073-4425/10/6/417/s1. Table S1: Number of RNA genes in the *Hypomyces perniciosus* genome. Table S2: Detailed classification of identifed repeat elements in *Hypomyces perniciosus* genome. Table S3: Genome features and *background* information of *Hypomyces perniciosus* and other representative fungi analyzed in this study. Table S4: Gene family contraction and expansion analysis results. Table S5: Annotation of positive selection genes during the evolution of *Hypomyces perniciosus*. Table S6: List of carbohydrate-active enzyme genes in Hypomyces perniciosus secretome. Table S7: (A) Total number of carbohydrate-active enzymes in *Hypomyces perniciosus* genome compared to other eight fungi used in this study. (B) Detailed list of carbohydrate-active enzymes modules in *Hypomyces perniciosus* genome compared to other eight fungi used in this study. Table S8: (A) Total number of antiSMASH secondary metabolites gene clusters in *Hypomyces perniciosus* genome. (B) The number of antiSMASH secondary metabolites gene clusters in *Hypomyces perniciosus* with known annotation. Table S9: Total number of NapDoS secondary metabolites genes in *Hypomyces perniciosus* genome. Table S10: The pathogen-host interaction (PHI) genes and annotation found in *Hypomyces perniciosus* genome. Table S11: List of membrane transport proteins in *Hypomyces perciosus* genome from Transporter Classification Database (TCDB). Table S12: List of fungal virulence factors genes in *Hypomyces* perciosus genome from database of fungal virulence factors (DFVF). Table S13: List of secretory protein genes in *Hypomyces perniciosus* genome. Table S14: List of effector protein genes from the secretome of *Hypomyces perniciosus* genome. Table S15: List of protease genes in *Hypomyces perniciosus* genome from MEROPS database. Table S16: List of Cytochromes P450 genes in *Hypomyces perniciosus genome*. Figure S1: Venn diagram representing the annotation of *Hypomyces perniciosus HP10* in five different databases. Figure S2: Number of genes.
