# Supplementary material for: Genome Analysis of Hypomyces perniciosus, the Causal Agent of Wet Bubble Disease of Button Mushroom (Agaricus bisporus)
Source: Genes (Basel). 2019 May 29;10(6):417. doi: 10.3390/genes10060417 (PMC6627653; doi:10.3390/genes10060417)
Supplement: Supplementary file 1 [file genes-10-00417-s001.zip › genes-489343-supplementary/Supplementary Figure S2.pdf]

Supplementary Figure S2

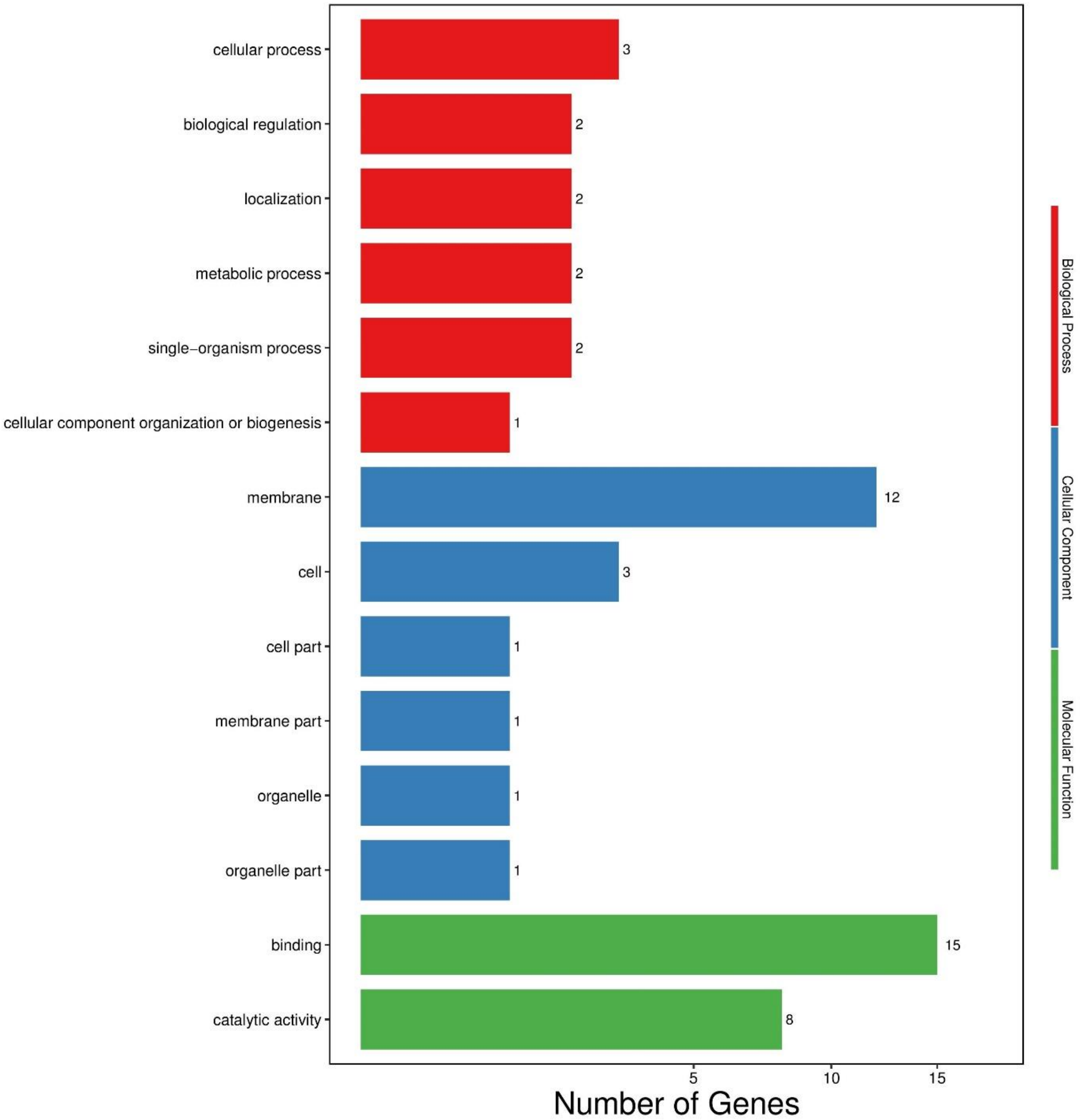

Figure S2: Gene Ontology (GO) classification of the positive selection gene in *Hypomyces perniciosus* HP10
